# Small Peptide Derivatives Within the Carbohydrate Recognition Domain of SP-A2 Modulate Asthma Outcomes in Mouse Models and Human Cells

**DOI:** 10.3389/fimmu.2022.900022

**Published:** 2022-07-08

**Authors:** Dave Francisco, Ying Wang, Craig Marshall, Michelle Conway, Kenneth J. Addison, Dean Billheimer, Hiroki Kimura, Mari Numata, Hong W. Chu, Dennis R. Voelker, Monica Kraft, Julie G. Ledford

**Affiliations:** ^1^Department of Medicine, University of Arizona, Tucson, AZ, United States; ^2^Asthma and Airway Disease Research Center, University of Arizona Health Sciences, Tucson, AZ, United States; ^3^Department of Medicine, National Jewish Health, Denver, CO, United States; ^4^Department of Cellular and Molecular Medicine, University of Arizona, Tucson, AZ, United States

**Keywords:** asthma, surfactant protein, SP-A, SP-A peptides, genetics

## Abstract

Surfactant Protein-A (SP-A) is an innate immune modulator that regulates a variety of pulmonary host defense functions. We have shown that SP-A is dysfunctional in asthma, which could be partly due to genetic heterogeneity. In mouse models and primary bronchial epithelial cells from asthmatic participants, we evaluated the functional significance of a particular single nucleotide polymorphism of SP-A2, which results in an amino acid substitution at position 223 from glutamine (Q) to lysine (K) within the carbohydrate recognition domain (CRD). We found that SP-A 223Q humanized mice had greater protection from inflammation and mucin production after IL-13 exposure as compared to SP-A-2 223K mice. Likewise, asthmatic participants with two copies the major 223Q allele demonstrated better lung function and asthma control as compared to asthmatic participants with two copies of the minor SP-A 223K allele. In primary bronchial epithelial cells from asthmatic participants, full-length recombinant SP-A 223Q was more effective at reducing IL-13-induced MUC5AC gene expression compared to SP-A 223K. Given this activity, we developed 10 and 20 amino acid peptides of SP-A2 spanning position 223Q. We show that the SP-A 223Q peptides reduce eosinophilic inflammation, mucin production and airways hyperresponsiveness in a house dust mite model of asthma, protect from lung function decline during an IL-13 challenge model in mice, and decrease IL-13-induced MUC5AC gene expression in primary airway epithelial cells from asthmatic participants. These results suggest that position 223 within the CRD of SP-A2 may modulate several outcomes relevant to asthma, and that short peptides of SP-A2 retain anti-inflammatory properties similar to that of the endogenous protein.

## Introduction

Surfactant Protein A (SP-A) belongs to a class of macromolecules produced in the lung known as collectins and possesses host defense and innate immune properties (1). We have shown that SP-A also has specific anti-inflammatory properties relevant to asthma and can be dysfunctional or deficient (2, 3). Specifically, we have shown that SP-A derived from asthmatic participants is defective in reducing *Mycoplasma pneumoniae-*induced inflammation and mucin gene expression (2). From our group, Lugogo et al. showed that SP-A was significantly decreased in asthmatic participants who were obese and that this correlated with worse lung function and asthma status (3). Recently, we have shown that SP-A can mediate Interleukin (IL)-13 induced allergic inflammation in both *in vitro* primary human lung epithelial cells and *in vivo* mouse models (4).

SP-A is a hydrophilic calcium-dependent (C-type) lectin produced in the lung epithelium of the distal airways, club cells and mucosal cells (5). A complete SP-A protein consists of a trimeric assembly of two molecules, SP-A1 and SP-A2, which oligomerize into an octadecamer possessing six trimers (6). Although SP-A1 and SP-A2 share approximately 96% amino acid sequence homology, the latter is believed to be more biologically active (7, 8). Wang et al. demonstrated that asthmatic participants have higher levels of SP-A1/total SP-A compared to healthy individuals, suggesting a reduction in SP-A2 or dysfunction with this protein may correlate with increased disease pathogenesis (2).

A single monomer of SP-A2, encoded by the SFTPA2 gene, is 248 amino acids in length and structurally composed of 4 domains: an N terminal sequence, collagen-like domain, neck-region, and a carbohydrate recognition domain (CRD) (9). In particular, the CRD recognizes oligosaccharide motifs and encourages the binding and opsonization of pathogens. Allelic variations corresponding to the CRD of SP-A2 have been associated with several respiratory diseases including tuberculosis, allergic rhinitis, respiratory syncytial virus, and influenza (10).

We identified a single nucleotide polymorphism (SNP) of SP-A2 (rs1965708) present in some of our asthma patients, which results in an amino acid substitution at position 223 and replaces a glutamine (Q) for a lysine (K) in the CRD. Our studies show that asthmatic individuals who possess two copies of the minor SP-A 223K allele are more likely to have lower lung function and worse asthma control as compared to asthmatic participants with two copies of the major SP-A 223Q allele.

Furthermore, *ex vivo* studies using primary bronchial epithelial cells show that exogenous SP-A2 223Q reduces IL-13-induced mucin gene expression more effectively than the SP-A 223K protein. To gain further insight into the importance of the major allele, SP-A223 Q, 10 and 20 amino acid peptides containing this region of interest were developed and studied using *in vitro* primary human lung epithelial cells and *in vivo* mouse models of asthma. We demonstrate that these truncated peptides retain anti-inflammatory properties similar to that of the full-length endogenous SP-A protein and may have potential therapeutic benefits in asthma.

## Materials and Methods

### Study Approvals

The Duke University and University of Arizona Institutional Review Boards (IRB) approved all protocols for recruitment of participants for these human studies. All animal experiments were carried out according to IACUC approved protocols at both Duke University and the University of Arizona.

### Human Studies

#### Description of Research Participants

Participants were recruited from the population in Durham, North Carolina, Tucson, Arizona and their surrounding areas. Informed consent was obtained from each participant (18-65 years of age). Asthmatic participants met criteria for mild and moderate asthma per GINA guidelines (11), including the presence of reversibility of airflow obstruction or airways responsiveness with a provocative concentration of methacholine resulting in a 20% fall in FEV_1_ (PC_20_ FEV_1_) of ≤ 8mg/ml or < 16 mg/ml if they were taking inhaled corticosteroids. The presence of atopy was determined using skin testing and peripheral eosinophils and were measured in a subset of participants. Healthy participants had no evidence of airflow obstruction, and no history of pulmonary disease. Exclusion criteria included an exacerbation of asthma within four weeks of study requiring antibiotics and/or corticosteroids, greater than 10-pack year history of tobacco use or any cigarette use in the last year, and any other significant medical conditions.

#### Research Bronchoscopy and Cell Culture

Participants underwent bronchoscopy with endobronchial-protected brushing and bronchoalveolar lavage, as previously described (12). The brushing of the proximal airways to obtain bronchial epithelial cells was performed under direct visualization using a separate protected cytologic brush for each pass, for a total of eight passes. Bronchoalveolar lavage (BAL) was performed *via* instillation of warm sterile saline in 60-ml aliquots, with return *via* gentle hand suction, for a total of 300 ml. Participants were discharged when their FEV_1_ achieved 90% of their pre-bronchoscopy, post-albuterol value. Freshly isolated airway bronchial epithelial cells from endobronchial brushing were cultured with BEGM (Lonza, Walkersville, MD) as previously described (12). Culture media contained the following supplements: Bovine pituitary extract, Insulin, Gentamycin-amphotericin, Retinoic Acid, Transferrin, Triiodothyronine, Epinephrine, and human Epidermal Growth Factor. After reaching confluence, cells were trypsinized and seeded onto collagen-coated polyester Transwell insert membranes of 12-mm diameter, at a concentration of 4 × 10^4^/well. Then the cells were cultured at air–liquid interface for 2 weeks to allow for differentiation.

#### SP-A Preparation

SP-A was purified from the BAL fluid of patients with alveolar proteinosis that were seen at Duke University Medical Center and were under IRB approval using previously described methods (13). Extracted SP-A was passed over a polymyxin B-agarose column to reduce endotoxin contamination and had final endotoxin concentrations of <0.01pg/mg SP-A as determined by the Limulus amoebocyte lysate assay [QCL-1000, BioWhittaker (Lonza)]. Recombinant SP-A was expressed in Freestyle HEK-293 cells and purified as previously described (14). This affinity purification procedure, which requires the C-type lectin activity of the protein be functional, ensures that the purified SP-A is properly folded. Small peptides were purchased from Genscript (Piscataway, NJ) and were received as lyophilized powder. All peptides were resuspended under sterile conditions in PCR-grade H_2_O. Peptide purity was greater than 95% and preparations were tested to confirm they were endotoxin-free. The SP-A 20-mer peptide sequence: PAGRGKEQCVEMYTDGQWND; the SP-A 10-mer peptide sequence: KEQCVEMYTD.

#### Sequencing of hSP-A2 Gene

Genomic DNA was extracted from peripheral blood using a PAXgene Blood DNA kit (Qiagen) according to manufacturer’s instructions. The region of interest containing SP-A2 coding sequence was assessed through allelic discrimination using TaqMan™ probes labeled with either HEX (encoding 223Q) or FAM (encoding 223K).

#### SP-A Binding Assay

Plates were coated with 100 ng/well of rIL-13 in PBS and placed at 4°C overnight. Wells were blocked with 5% non-fat milk in PBS/1% Triton X-100 for 2 hrs at 37°C after which purified samples of SP-A (1 mg/ml) were added in blocking buffer and incubated for an additional 2 hrs at 37°C. The plate was washed 5 times with PBS/1% Triton X-100 and HRP conjugated anti-human SP-A was added (1:2000) in blocking buffer for 1 hr at 37°C, after which the plate was washed an additional 5 times as described above. Finally, orthophenylene diamine substrate was added for 5 min, after which stop solution was added and samples were assessed using an ELISA plate reader at absorbance of 490 nm.

#### Western Analysis of STAT Activation

Human bronchial epithelial cells were grown on transwell inserts at an air-liquid interface for two weeks prior to experimentation. Some wells received chronic IL-13 challenge (10 ng/ml each day for 5 days on the apical surface) in the presence of absence of SP-A (20 μg/ml, 30 min prior to IL-13 on the apical surface). For collection of human epithelial cells or mouse lungs, radioimmunoprecipitation assay (RIPA) buffer (Millipore) with protease inhibitors (Roche) were added to each sample for cell lysis or tissue homogenization. Each sample was homogenized, sonicated, and insoluble material was removed by centrifugation. The protein concentration of each lysate was determined by BCA and an equal amount of lysate was loaded onto electrophoretic gels for each sample to be analyzed by Western. Antibodies for phospho-Stat3, Stat3 and β-actin were all used according to manufacturer’s (Cell Signaling) recommendations and analyzed in the order listed respectively for each blot.

#### Statistical Analysis

For human data analysis, associations between SPA2 SNP rs1965708 and asthma status were evaluated using Fisher’s exact test, and separately, logistic regression with the number of “A” alleles as an explanatory variable. Lung function measurements (FEV_1_, FVC, and FEV_1_/FVC ratio), blood cell types (eosinophils, neutrophils) and BAL cell types (macrophages, eosinophils, neutrophils, epithelial cells) were evaluated using linear models with asthma status and SPA2 SNP genotype as categorical explanatory variables. Because no non-asthmatic participants with AA genotype have blood or BAL cell type data, these comparisons combined the heterozygote AC and homozygote AA SPA2 genotypes into a single category. Analysis of variance was used to assess asthma and SPA2 SNP factors, as well as their interaction. Tukey’s HSD is used for pairwise comparisons. Note that Tukey’s procedure accounts for all pairwise comparisons within a single analysis in computing p-values. P-values less than 0.05 were considered statistically significant, but no adjustment was made for testing the multiple related outcomes in these exploratory clinical data. Airways hyperresponsiveness, as measured by the provocative concentration of methacholine resulting in a 20% fall in FEV1 (PC20 FEV1) was compared between the AC and CC asthmatic groups using Student’s t test of the logPC20 FEV1.

MUC5AC expression fold-change, relative to control, was computed for IL-13 alone, IL-13 + AA10, and IL-13 + AA20. We used log10 transformed data to reduce skewness, and a linear mixed model for statistical analysis. The mixed model extends the usual analysis of variance model to reflect the ALI intra-subject treatments with IL-13 + SP-A peptides, and the inter-subject asthma vs. control groups. Inference proceeded hierarchically, first requiring main effects or interactions be significant (p < 0.05) before proceeding to *post hoc* comparisons. Tukey’s honestly significant difference was used for pairwise comparison of groups with a significance level of 0.05.

### Mouse Studies

#### Description of Mice

SP-A humanized transgenic mice were generated as previously described (15). SP-A^-/-^ mice on C57BL/6 background (16) were bred in-house and wild-type (WT) mice were purchased from Jackson Laboratories (Bar Harbor, ME) and bred in house for experiments. Age-matched (approximately 6-8 weeks) male mice were used for experiments since they have more robust methacholine sensitivity for lung function measurements compared to female mice. Mice were anesthetized under inhaled isoflurane and given 3.9 μg of recombinant IL-13 (Peprotech) in 50 μl of sterile saline *via* oropharyngeal delivery (17). At the desired time point, mice were euthanized and the lungs lavaged with PBS (0.1 mM EDTA) and lung tissue was obtained for further analysis. Differential cell counts were analyzed from the lavage fluid after H&E staining. Viability was assessed by Trypan blue exclusion.

#### House Dust-Mite Model

The common allergic airway model was used in which 6-8 weeks old C57BL/6 male mice were sensitized and challenged with 100 μg house-dust mite extract (HDM; prepared from dry weight, Greer) over the course of three intranasal instillations while under isoflurane anesthesia. Mice received either vehicle, SP-A 10-mer or 20-mer peptides 24 hrs after HDM challenge *via* oropharyngeal delivery while under isofluorane anesthesia.

On the day of pulmonary function testing (PFT), mice were anesthetized with urethane (Sigma, U2500) prepared at a concentration of 125 mg/ml in sterile dH2O and dosed at 16 μl/gram of body weight intraperitoneally (ip). Once a surgical plane of anesthesia is reached, the trachea was cannulated with a 19-gauge metal cannula using previously described methods (18, 19). Mice were connected to a commercial computer-controlled piston-ventilator (flexiVent, SCIREQ Inc., Montreal, Qc, Canada) for mechanical ventilation and PFTs (19). Pancuronium bromide (0.8 mg/ml in saline, Sigma P1918) was administered to the anesthetized mouse *via* ip injection at a volume of 10 μl/gram of body weight to prevent any interference from the subject during the PFTs. Following an equilibration period under default mechanical ventilation settings (150 breaths/min, tidal volume of 10 ml/kg, and a PEEP of 3 cmH_2_O), two recruitment maneuvers (inflation to a standard pressure of 30 cmH_2_O over a 3 second and holding for an additional 3 seconds) were performed to open and close lung areas and to standardize lung volume history. Methacholine challenges were carried out as previously described (20). Mice that were not subject to PFTs were euthanized and the lung lavaged with PBS (0.1 mM EDTA) and lung tissue was obtained for further analysis. Differential cells counts were analyzed from the lavage fluid after H&E staining. Viability was assessed by Trypan blue exclusion.

#### RT-PCR

Mouse tissues and human bronchial epithelial cells were collected into 1 ml of TRI Reagent^®^ (Sigma). RNA was isolated using the standard TRI reagent/chloroform extraction method. DNA was synthesized from 1 µg of total RNA using Bio-Rad™ cDNA Synthesis kit. Real-time polymerase chain reaction (RT-PCR) was performed using Bioline 2x SensiFAST SYBR no-ROX mix. The samples were analyzed for expression levels of mouse MUC5AC using forward and reverse primers specific to the gene (forward 5’ GAG GGC CCA GTG AGC ATC TCC 3’, reverse 5’ TGG GAC AGC AGC AGT ATT CAG T 3’). The relative levels of expression obtained were normalized to the mammalian housekeeper gene Cyclophilin using primers specific to the gene (forward 5’ AGC ACT GGA GAG AAA GGA TTT GG 3’, reverse 5’ TCT TCT TGC TGG TCT TGC CAT T 3’). For human bronchial epithelial cells, each condition is compared to its unexposed negative control after standardization to the housekeeper gene, GAPDH.

#### Histological Analysis

Mice were euthanized by CO_2_ asphyxiation. Left lung lobes were dissected and immersed in 10% buffered formalin for fixation. After 3 days, the lung lobes were transferred from formalin to 70% ethanol, then routinely processed and paraffin embedded for PAS staining to assess for mucin production. Stained sections were scored blinded according to standard methods (21).

#### Statistical Analysis

For murine experiments, analyses were performed using Graph Pad Prism and Student’s t-Test or one-way ANOVA, as necessary. F-test was used to compare variances between data analyzed and Welch’s correction applied if variances were significantly different (p<0.05) between groups statistical significance was defined as a p-value < 0.05.

## Results

### Humans: Genetic Variation in SP-A2 is Associated With Changes in Lung Function

Seventy-five participants with mild-moderate asthma (FEV_1_: 87 ± 2% predicted), and sixty-eight non-asthmatic participants (FEV_1_: 100 ± 2% predicted) underwent blood sampling for gene sequencing of the SP-A2 gene (**Table 1**). By Fisher’s exact test, the SP-A2 SNP associated with asthma status (p=0.023), with the “A” allele (Lysine/K at position 223) associated with greater odds of disease. Logistic regression with number of “A” as a factor indicates increasing odds of disease (ChiSq=7.5, df=2, p=0.023) with the minor allele. While there is an association of the minor allele and asthma status, there does not appear to be an association with increased reported disease severity (p=0.65).

**Table 1:** Demographics for patients undergoing asthma phenotyping and blood genotyping for SP-A2.

rs1965708 SFTPA2	AA		AC		CC	
	Asthma	Normal	Asthma	Normal	Asthma	Normal
**Asthma/Normal**	8	1	35	30	32	37
Males**	3	0	11	8	11	12
Females**	5	1	24	21	21	25
**White**	1	0	17	14	21	24
**Black**	5	1	16	14	8	10
**Hispanic**	0	0	0	0	2	1
**Asian**	2	0	2	2	1	2
Age*	36 ± 5.2	28	30 ± 1.6	31 ± 1.9	29 ± 2	29 ± 1.5
FEV1 (%)*	72.62 ± 3.4^†^	109	91.17 ± 2.2^†^	101.89 ± 2.6	87.77 ± 2.2	99.75 ± 1.9
FVC (%)*	81.62 ± 3.7¥	117	99.5 ± 2.2¥	105.92 ± 3.1	98.83 ± 1.6¥	101.55 ± 2.0
FEV1/FVC (%)*	73.37 ± 1.9	80	75.62 ± 1.5	81.82 ± 1.3	75.09 ± 1.5	83.61 ± 1.1
PC20 (mg/ml)*	0.63 ± 0.35	16	1.23 ± 0.3	16.34 ± 0.3	0.91 ± 0.2	16
ACQ*	2.82 ± 0.2	N/A	1.19 ± 0.2	N/A	1.59 ± 0.1	N/A
Exacerbations per year*	1	N/A	1.14 ± 0.1	N/A	0.75 ± 5	N/A
**Atopy (% positive)**	75	0	93	37	84	47
**Total Peripheral Eosinophils**	N/A	N/A	1.78 x 10^10 ± 1	8.37 x 10^9 ± 0.8	3.25 X 10^9 ± 1.3	1.9^10^9 ± 0.4
**Total Peripheral Neutrophils**	N/A	N/A	2.58 x 10^11 ± 0.5	3.27 x 10^11 ± 0.5	6.29 X 10^10 ± 0.3	5.02 x 10^10 ± 0.5
**Medications**						
**Short acting beta agonist**	7	N/A	33	N/A	29	N/A
**Leukotriene modifier**	2	N/A	2	N/A	3	N/A
**Inhaled corticosteroid (ICS)**	1	N/A	3	N/A	2	N/A
**Antihistamine**	3	N/A	10	N/A	11	N/A
**Combination therapy (ICS/LABA)**	3	N/A	5	N/A	5	N/A
**Short acting anticholinergic**	1	N/A	N/A	N/A	N/A	N/A

*Mean ± SE; **One participant did not specify gender.

^†^p = 0.002 AA < AC; ¥ p< 0.05; AA < AC and CC. N/A, not applicable.

In addition, there is an association between SP-A2 SNP, asthma status and lung function (FEV_1_%, FVC% and FEV_1_/FVC ratio). This effect may be modified with combined modeling of subjects’ SP-A2 SNP and asthma status. FEV_1_ (% predicted) is associated with both asthma status (p<0.001) and SP-A2 SNP (p=0.003), with a possible interaction (p=0.16). Pairwise group comparisons indicated that among asthmatics, the AA genotype (encodes for 223K/K) exhibits reduced FEV_1_ (% predicted) with an approximately 16% reduction compared with CC (223Q/Q, p=0.057) and AC (p=0.002), respectively. Among non-asthmatics, significant differences in lung function are not demonstrated between genotypes. FVC (% predicted) is associated with both asthma status (p<0.003) and SP-A2 SNP (p=0.003), with a possible interaction (p=0.08). Pairwise group comparisons indicated that among asthmatics, the AA genotype exhibits reduced FVC (about 17%) compared with CC (p=0.021) and AC (p=0.003), respectively. Among non-asthmatics, strong differences are not indicated. The FEV_1_/FVC ratio is associated with asthma status (p<0.001), but not with the SP-A2 SNP. The ratios average 0.07 lower for participants with asthma as compared to non-asthma. Within the group who underwent bronchoscopy (**Table 2**), the methacholine PC20 FEV1 was significantly lower in the AC asthma group compared to CC asthma group, suggesting greater hyperresponsiveness in the AC asthmatic group (p=0.03)

**Table 2:** Demographics for patients that underwent bronchoscopy for analysis of bronchial epithelial cells.

rs1965708 SFTPA2	AC		CC	
	Asthma	Normal	Asthma	Normal
**Asthma/Normal**	4	4	7	5
**Males**	3	1	1	2
**Females**	1	3	6	3
**White**	0	2	4	3
**Black**	4	2	2	2
**Hispanic**	0	0	0	0
**Asian**	0	0	1	0
Age*	24 ± 2.3	35 ± 6.1	26 ± 2.2	30 ± 1.4
**FEV1 (%)**	90.5 ± 6.0	100.8 ± 9.0	88.3 ± 4.2	101.5 ± 2.4
**FVC (%)**	99.5 ± 6.0	99.75 ± 8.3	101.3 ± 3.0	100 ± 2.5
**FEV1/FVC (%)**	77.0 ± 3.4	83.0 ± 1.6	74.4 ± 2.7	84.3 ± 2.6
**PC20 (mg/ml)**	0.5 ± 0.2^┼^	N/A	1.8 ± 0.4^┼^	N/A
**ACQ**	1.5 ± 0.2	N/A	1.3 ± 0.1	N/A
**Exacerbations per year**	1.5 ± 0.5	N/A	1	N/A
**Atopy (% positive)**	100	25	83	60
**Total Eosinophils**	2.7 x 10^10 ± 1.5	9 x 10^9 ± 3.9	2.5 x 10^10 ± 1	N/A
**Total Neutrophils**	3.5 x 10^11 ± .48	4.5 x 10^11 ± 1.5	2.9 x 10^11 ± .3	N/A
**Medications**				
**Short acting beta agonist**	4	N/A	7	N/A
**Leukotriene modifier**	N/A	N/A	N/A	N/A
**Inhaled corticosteroid (ICS)**	N/A	N/A	N/A	N/A
**Antihistamine**	N/A	N/A	3	N/A
**Combination therapy (ICS/LABA)**	N/A	N/A	1	N/A
**Short acting anticholinergic**	N/A	N/A	N/A	N/A

*Mean ± SE; ^┼^Significant p<0.05. N/A, not applicable.

A subset of the SP-A2 genotyped asthmatic participants was further analyzed for inflammatory cells in the peripheral blood. Due to the relatively small number of homozygous 223K individuals carrying the minor allele AA which translates to the amino acids KK, participants carrying one (223Q/K) or two copies (223K/K) of the minor allele were grouped for analysis. Blood eosinophils were increased in participants with asthma (p=0.002), but no significant association was observed in our cohort for blood neutrophils. There was no significant association between the SP-A2 SNP and blood eosinophils or neutrophils.

### Mice: SP-A2 Genetic Variation Influences Protection Against IL-13

In order to determine the consequence of SP-A2 223Q and 223K variations *in vivo*, we examined the response to IL-13 challenge in humanized SP-A2 transgenic mice. These mice are devoid of mouse SP-A and express either the human version of SP-A2 223Q or SP-A 223K at similar RNA gene expression and protein levels, as described previously (15). There were no gross differences in the humanized SP-A transgenic mice prior to challenge and saline treated controls had similar levels of BAL cells across all genotypes of mice examined which consisted of >95% macrophages (**Figure S1**).

**Figure 1:**
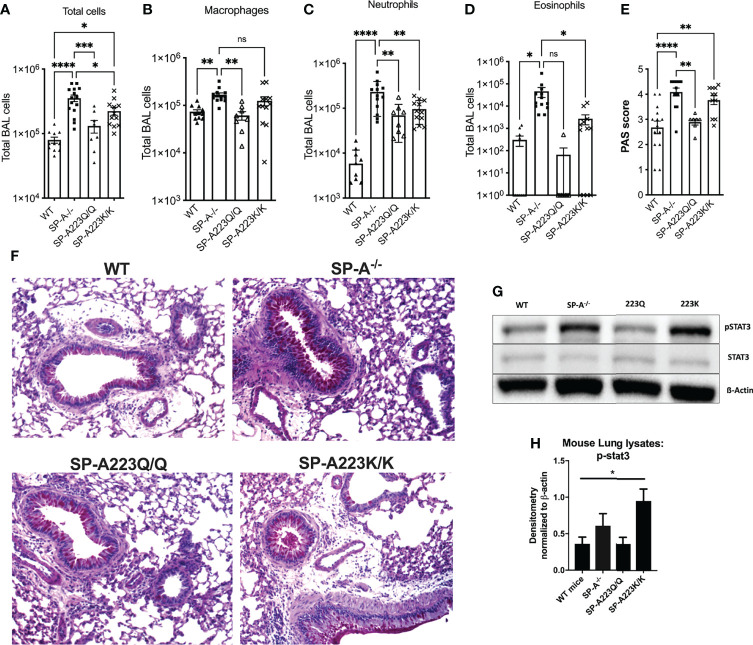
Genetic variation in SP-A2 determines extent of protection against IL-13 induced inflammation in humanized SP-A transgenic mice. BAL cells from **(A)** IL-13 challenged mice, which consisted of and **(B)** macrophages, **(C)** neutrophils and **(D)** eosinophils. **(E)** PAS scored lung histology from IL-13 challenged mice. N=12 WT; 15 SP-A^-/-^; 8 SP-A223Q/Q; 12 SP-A223K/K per group from 3 separate experimental repeats. *p < 0.05, **p < 0.01, ***p < 0.001. ****p < 0.0001 by One-way Anova with Dunnett’s test for multiple comparisons. **(F)** Representative PAS images of each genotype treated with IL-13. **(G, H)** Stat3 phosphorylation by Western blot and densitometry from representative lung samples; *p<0.05 by One-Way Anova with Tukey’s multiple comparisons.

When challenged with IL-13, there were significantly more total cells in BAL of SP-A^-/-^ mice as compared to WT mice (**Figure 1A**), which was contributed by increases in macrophages, neutrophils and eosinophils (**Figures 1B–D**). However, mice that were homozygous for human 223Q had significantly decreased total BAL cells (**Figure 1A**) as compared to SP-A^-/-^ mice; the differential counts were not significantly different from WT mice (**Figures 1B–D**). In contrast, mice homozygous for 223K, had less protection as defined by total cells in BAL when compared to SP-A^-/-^ mice and 223Q/Q mice.

After IL-13 challenge, the 223Q-expressing mice had significantly less mucin production as compared to SP-A^-/-^ mice and were similar to WT mice (**Figures 1E, F**). There were no differences in mucin production in the saline control mice across the different genotypes (data not shown). Since we had previously determined that SP-A regulated Stat3 phosphorylation in response to IL-13 (4), we next wanted to assess whether there were differences in Stat3 phosphorylation in the lung tissue of the humanized mice challenged with IL-13. As shown in **Figures 1F, G**, there was significantly more Stat3 phosphorylation in lungs from IL-13 challenged 223K mice as compared to WT mice. There was no discernable difference in Stat3 phosphorylation when comparing 223Q mice to WT mice.

### Humans: SP-A2 Genetic Variation Modulates Protection Against IL-13 in Epithelial Cells

**Figure 2:**
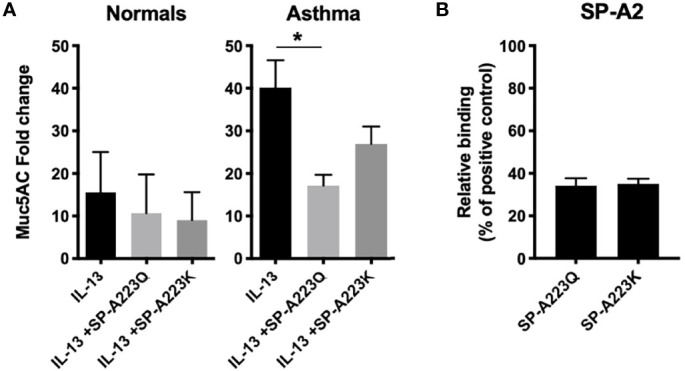
Genetic variation in SP-A2 determines extent of protection against IL-13-induced inflammation from bronchial epithelial cells from asthmatic participants. **(A)** MUC5AC RNA expression from bronchial epithelial cells (n=3 normal, n=3 asthma) grown at ALI and treated with IL-13 for 5 days in the presence of absence of full-length recombinant SP-A2(223K) (20 μg/ml) or SP-A2(223Q) (20 μg/ml) that were added 30 min prior to challenge. After standardization to the housekeeper gene, data are displayed as fold relative to the non-IL-13 challenged control for each respective patient set, with the standard deviation shown. Average fold change and standard deviation are displayed. *p < 0.05. **(B)** Genetic variants of SP-A2 that differ only at position 223 (Q and K) were examined for relative binding to IL-13 relative to extracted human oligomeric control SP-A.

Previously, we have shown that SP-A extracted from participants with mild asthma is dysfunctional in the immune regulation of pathogens (2). One possibility for defective function of SP-A in asthma is genetic variation in the setting of type 2 inflammation. Since our clinical data showed an association between the SP-A2 SNP, asthma status and lung function, we further tested the ability of two SP-A recombinant genetic variants (full-length containing either 223Q or 223K) to suppress inflammation to IL-13 *ex vivo.* We used an affinity purification procedure, which requires the C-type lectin activity of the protein be functional, thus ensuring that we are purifying properly folded and active forms of SP-A. We observed minimal responses from IL-13 challenged cells derived from normal participants (**Figure 2A**). However, IL-13 challenged bronchial epithelial cells from a separate cohort of asthmatic participants (see **Table 2** for demographics) resulted in an approximately 30-fold increase in MUC5AC when the cells were exposed over the course of 5 days. When co-cultured with recombinant SP-A2 223Q at a physiologic concentration (20 μg/ml), MUC5AC expression was significantly attenuated in the IL-13 challenged/exposed cells derived from asthmatic participants (p=0.029), whereas the SP-A2 223K variant was not as effective (p=0.157) (**Figure 2A**). Further examination of common variants in SP-A2 at position 223 revealed that variants 223Q and 223K, which had different effects against MUC5AC, bound to IL-13 similarly (**Figure 2B**) suggesting the function of SP-A2 is independent of binding to IL-13.

### Mice: Development and *In Vivo* Testing of SP-A Peptides on Inflammation in an Asthma Model

**Figure 3:**
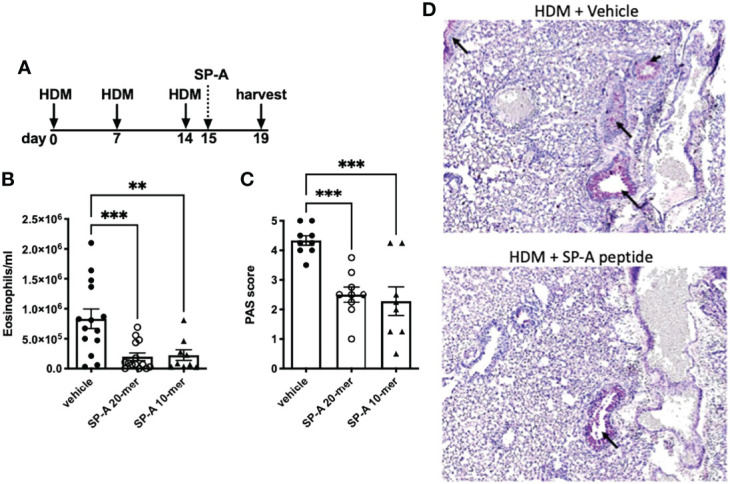
HDM-challenged SP-A deficient mice treated with SP-A peptides have reduced hallmarks of inflammation. **(A)** SP-A deficient mice challenged intra-nasally with HDM on days 0, 7, and 14. On day 15 mice were divided into groups and given either vehicle or SP-A peptides (10-mer or 20-mer) *via* oropharyngeal instillation at 25 μg/ml concentration (~1 mg/kg body weight). On day 19, mice were sacrificed and **(B)** eosinophils in the BAL and **(C)** mucin production in **(D)** lung histological sections were assessed. n = 10,10, **p < 0.01, ***p < 0.001 by One-way Anova for multiple comparisons.

The use of house dust-mite is a well-established model of allergic airways disease in mice that is known to induce recruitment of eosinophils, mucin production and enhance airways hyperresponsiveness. To test the SP-A 20-mer and 10-mer peptides on inflammation associated with allergic airways disease, SP-A deficient mice were challenged with HDM on days 0, 7 and 14 as described in **Figure 3A**. At day 15, when inflammation is at a maximum, 1 dose of SP-A peptides (20-mer or 10-mer, both containing Q from site 223) or vehicle were delivered. The timing of this model and dosing were based on our previous study in which full-length SP-A was given at the peak of eosinophils (24 hrs after the last challenge) and eosinophils assessed in BAL 4 days later (22). Eosinophils and mucin production were examined four days after peptide therapy as an indicator of inflammation. As demonstrated in **Figure 3B**, both the 20-mer and the 10-mer resulted in significantly decreased eosinophils in the BAL as compared to the vehicle treated control. There were no eosinophils detected in the BAL of control non-HDM challenged mice that received vehicle (**Figure S1**). In addition, treatment with either the 20-mer or the 10-mer led to significanlty reduced mucin production as detected in PAS stained lung histology (**Figures 3C, D**). There were no differences in mucin production in the non-HDM challenged mice that received vehicle or SP-A peptides (not shown).

### Mice: *In Vivo* Testing of SP-A Peptides on Airway Hyperresponsiveness in an Asthma Model

**Figure 4:**
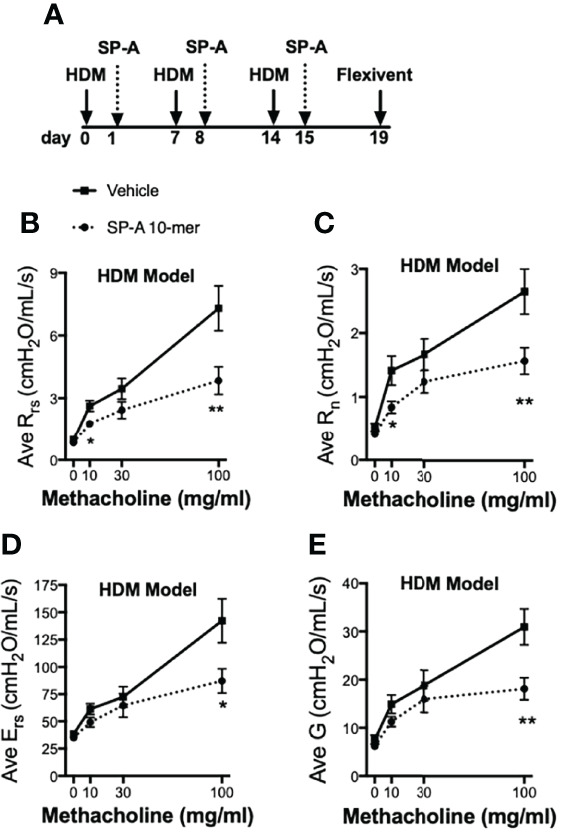
HDM-challenged WT mice treated with SP-A peptides have reduced sensitivity to methacholine challenge. **(A)** WT male mice challenged intra-nasally with HDM on days 0, 7, and 14. On days 1, 8 and 15 mice were divided into groups and given either vehicle or SP-A peptides (10-mer, 25 μg/ml, ~1 mg/kg body weight) *via* oropharyngeal instillation. On day 19, pulmonary function tests during a methacholine challenge were performed while mice were under anesthesia. **(B)** Total airways resistance (Rrs), **(C)** Newtonian resistance (Rn), **(D)** total airways Elastance (Ers) and **(E)** tissue damping **(G)** were assessed by flexivent. Data graphed are mean +/- SEM. n = 12,12, *p < 0.05, **p < 0.01 by t-test at each indicated dose.

In another set of experiments, pulmonary function tests (PFTs) during methacholine challenge were carried out in WT male mice and we chose to use the 10-mer peptide since we anticipate the smaller peptides will be easier to deliver with the use of inhalers in future drug development models. For these studies, mice were challenged with HDM on days 0, 7 and 14 as described in **Figure 4A**. Twenty four hours after each HDM challenge, mice received either vehicle or SP-A peptides (10-mer). We chose to deliver a dose of our test peptides after each HDM challenge in order to assess their impact on overall lung function and not merely clearance of eosinophils, which are but one of several contributors to the asthma phenotype. PFTs were assessed four days after the last SP-A treatment. HDM provoked an elevated response to methacholine compared to the negative control saline treated mice (**Figure S1**). The resistance of the respiratory system (R_rs_), which reflects the overall airflow resistance at the breathing frequency, was significantly decreased in the SP-A peptide treatment group as compared to vehicle control group (**Figure 4B**). This measurement includes a contribution from the conducting and peripheral airways, the tissue, and the chest wall. In addition, the Newtonian resistance (R_n_), which is dominated by the resistance of the large conducting airways not involved in gas exchange, was also significantly reduced in the peptide treated group as compared to the vehicle treated group (**Figure 4C**).

The respiratory system elastance (E_rs_), which quantifies the overall stiffness of the entire respiratory system during tidal breathing was also significantly reduced at the highest doses of methacholine challenge in the peptide treated group as compared to the vehicle treated group (**Figure 4D**). Finally, tissue damping (G), which reflects a measure of the amount of energy that is lost within the tissues as a result of friction and includes resistance to air flow in the peripheral airways, was significantly reduced at the highest doses of methacholine challenge in the peptide treated group as compared to the vehicle treated group (**Figure 4E**).

### Mice: *In Vivo* Testing of SP-A Peptides on Pulmonary Function During Non-allergic IL-13 Challenge

**Figure 5:**
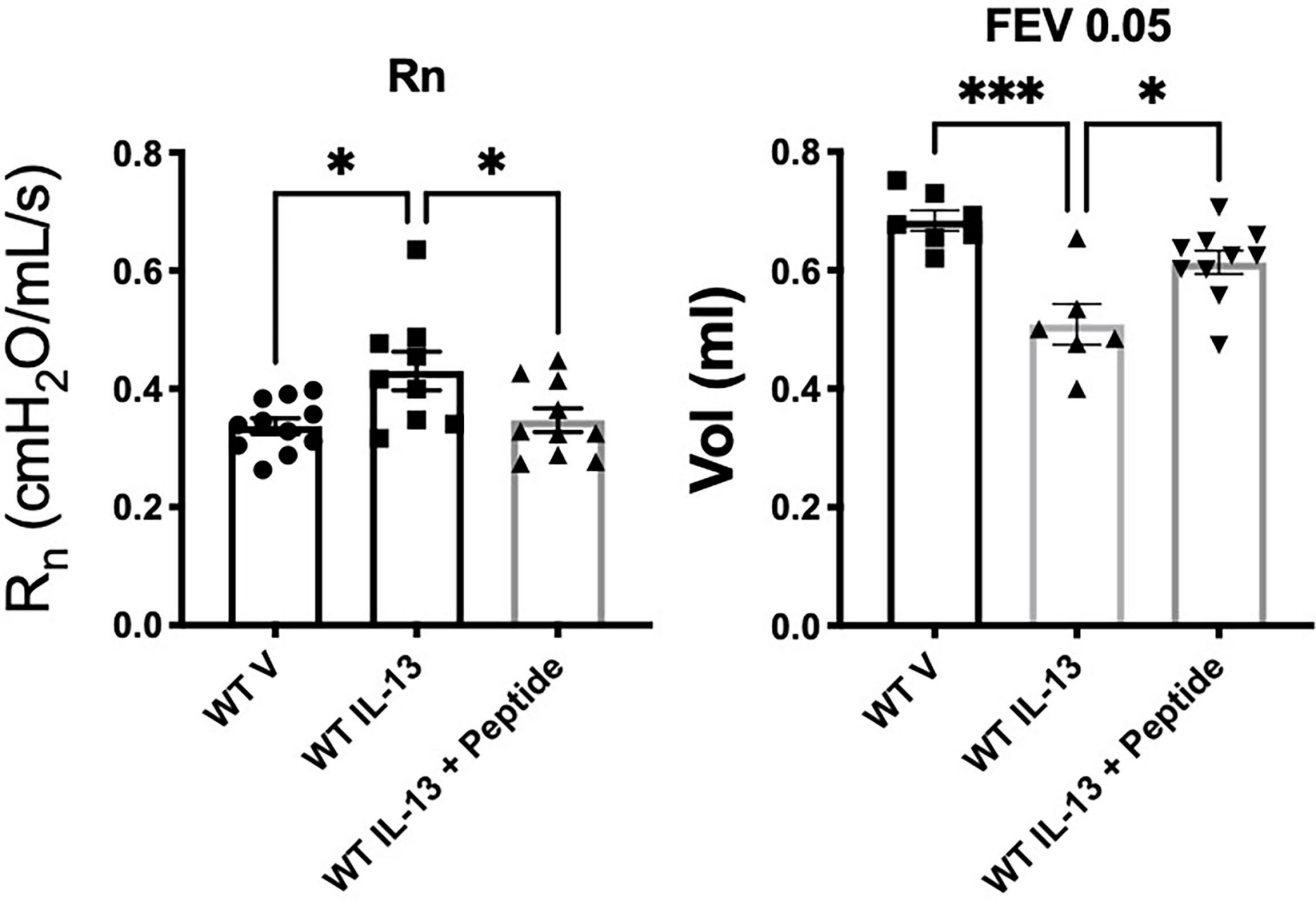
IL-13-challenged mice treated with SP-A 10-mer peptide have improved lung function. WT male mice were challenged with vehicle (saline) or IL-13 (3.9 μg) by oropharyngeal delivery for 3 consecutive days. Two hours after each IL-13 challenge, mice received either vehicle (saline) or SP-A 10-mer peptide (25 μg/ml; ~1mg/kg body weight) *via* oropharyngeal delivery. Pulmonary function tests were conducted on day 4 on a flexiVent machine (SCIREQ) with the negative pressure-driven forced expiration (NPFE) extension. IL-13 challenge resulted in significantly increased Newtonian resistance (Rn) and decreased forced expiratory volumes (FEV) at 0.05 seconds. Treatment with SP-A 10-mer peptide protected against IL-13 induced increase in Rn and decreases in FEV. Average mean +/- SEM is graphed, n= as shown from 2 independent experiments. *p < 0.05, ***p < 0.001 by ANOVA for multiple comparisons.

In order to test whether the SP-A 10-mer was effective in a non-allergic model of asthma, we tested the SP-A peptides (10-mer) in the IL-13 challenge model. For this, WT male mice were challenged with vehicle or IL-13 by oropharyngeal delivery for 3 consecutive days. Two hours after each IL-13 challenge, mice received either vehicle (saline) or SP-A 10-mer peptide (25 μg) also *via* oropharyngeal delivery. Pulmonary function tests were conducted on day 4 on a Flexivent machine (SCIREQ) with the negative pressure-driven forced expiration (NPFE) extension. Since IL-13 is such a strong inducer of lung function decline by non-allergic mechanisms, a methacholine challenge was not necessary to determine the effectiveness of SP-A peptides in this model. As shown in **Figure 5**, IL-13 challenge resulted in significantly increased Newtonian resistance (Rn) and decreased forced expiratory volumes (FEV) at 0.05, 0.1 and 0.2 seconds. Treatment with SP-A 10-mer peptide led to significant protection against IL-13-induced increase in Rn and decreases in FEV across all time points assessed.

### Humans: Small SP-A 20-mer Peptide Attenuates IL-13 Induced MUC5AC in Epithelial Cells From Asthma

**Figure 6:**
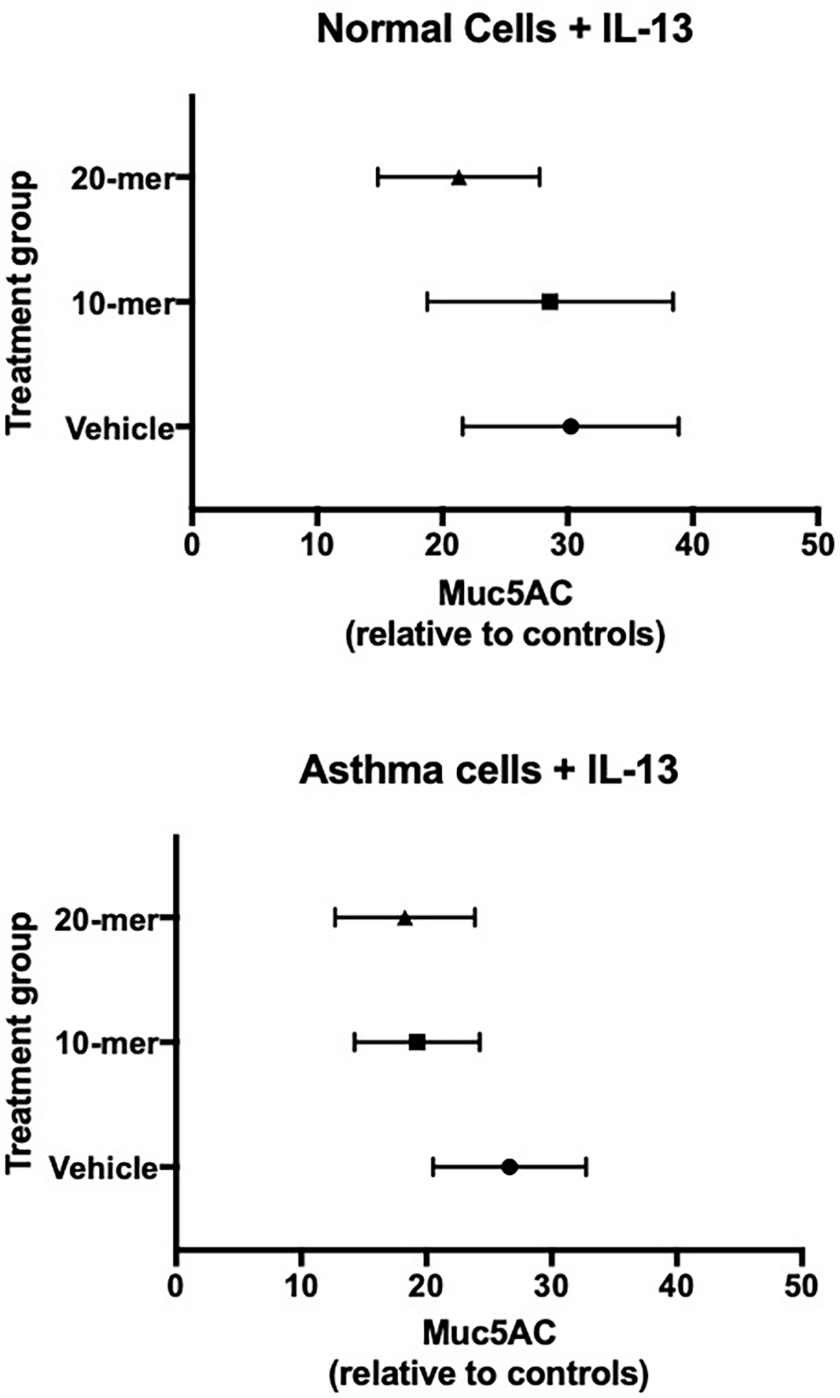
Primary human lung epithelial cells treated with SP-A peptides have reduced IL-13 induced MUC5AC gene expression. Primary human bronchial epithelial cells derived from normal and asthmatic participants were incubated for 30 minutes with either 20-mer or 10-mer SP-A peptide (20 μg/ml) before stimulation with IL-13 (10 ng/ml) for 5 days. When taken together (asthma and normal cells), MUC5AC gene expression was significantly reduced in the SP-A 20-mer treatment group compared with IL-13 alone (p=0.004).

Since our findings in asthmatic participants as well as mouse models indicated that SP-A2 expressing glutamine at position 223 was active in reducing type 2 inflammation, we sought to determine if our small peptides encompassing the 223 area of interest have activity in human cells from asthmatic participants. Based on our previous findings with 20 amino acid peptides spanning this region, we tested the 20-mer containing a glutamine at position 223 with surrounding amino acids identical to those in endogenous SP-A (23) and also a shortened 10-mer version. Bronchial epithelial cells obtained from normal and asthmatic participants were exposed to IL-13 in the presence of SP-A 20-mer, 10-mer or vehicle and assessed for MUC5AC by RT-PCR. Assessment of treatment (vehicle vs SP-A-mer) was done for the same set of patient cells and graphically displayed as a fold change over their respective control, which is the level of MUC5AC in a non-challenged control set of wells. As shown in **Figure 6**, MUC5AC gene expression was significantly reduced in asthmatic and normal participants with SP-A 20-mer treatment as compared to IL-13 with vehicle (p=0.004).

## Discussion

We recently published that SP-A decreases mucin secretion and inflammation in response to IL-13 challenge using a combination of SP-A deficient mice and primary human airway epithelial cells from participant with and without asthma (4). In mice lacking SP-A, we demonstrated that IL-13 significantly increased airway neutrophil and eosinophil recruitment and mucin production compared to WT mice. Likewise, phenotypes observed in human primary cells grown at an ALI and exposed to IL-13 were significantly reduced when SP-A was administered alongside IL-13 (4). Mechanistically, we showed that SP-A modulated IL-13-induced inflammation *via* mediating downstream IL-6/Stat3 signaling. While we have previously shown that SP-A demonstrates anti-inflammatory effects relevant to asthma, our new findings suggest there is genetic heterogeneity in these functions.

In this report, we first identified a polymorphism at position 223 of the CRD of SP-A2 peptide as relevant to asthma based upon human SP-A2 gene sequencing. Participants with two copies of the SNP rs1965708, which results in a substitution of a glutamine for a lysine at position 223 of SP-A2 peptide, were more likely to have lower lung function and worse asthma control, as discussed in the results section. To further investigate the functional significance of this SNP, we bred mice to express two copies of the major or minor allele of this SNP (15). Similar to what we observed in humans, the mice expressing two copies of the minor allele where a glutamine (Q/Q) was substituted for a lysine (K/K), these mice were not as protected from IL-13 challenge with increased inflammation compared to the mice expressing two copies of the major allele (Q/Q).

Recognizing the importance surrounding this amino acid change, we created 10- and 20-mer SP-A2 peptides that included position 223 from the CRD in order to determine if replacement of SP-A with a functional variant would modulate endpoints relevant to asthma. We chose this particular region of the CRD based on our findings in asthma patients as described above and based on our studies in which full-length SP-A that contains the Q at position 223 was able to induce eosinophil apoptosis, which was CRD-dependent, while SP-A containing K at position 223 was not active in this function, as we reported previously (22). Here we report that the CRD-containing peptides that included Q at position 223 were effective at reducing inflammation in SP-A deficient during HDM challenge, reducing AHR in WT mice following HDM challenge, and protecting from lung function decline in WT mice following a high dose IL-13 challenge. Lastly, we also show that the Q variant is more effective than the K variant at inhibiting Stat3 phosphorylation in SP-A deficient mice challenged with IL-13, suggesting a possible mechanism of action of the SP-A peptides.

Reports over the last decade have linked SP-A and asthma. Pastva and colleagues showed in 2011 that SP-A deficient mice sensitized and challenged with OVA manifested a robust type 2 inflammatory response with increased airway eosinophils, interleukin-4, 5 and IgE (24). Our group showed that SP-A extracted from bronchoalveolar lavage (BAL) from participants with asthma ineffectively reduced inflammation due to *M. pneumoniae* infection of airway epithelial cells compared to SP-A extracted from participants without asthma (2). Additionally, we reported that SP-A levels in BAL are reduced in asthmatic participants who are obese, and this reduction correlated with BMI and asthma status (3). These data, coupled with our recent report demonstrating inhibition of Stat3 phosphorylation by SP-A as a potential mechanism suggest that SP-A exhibits immune modulatory effects in asthma. We report here that the effects are not uniform across all genotypes of SP-A as K at position 223 of SP-A2 results in reduced phosphorylation of Stat3 as compared to the Q variant. These observations have allowed creation of a construct that replacement of SP-A with the more effective variant may be a possible treatment for asthma.

Our report has several limitations. It is certainly possible that other variants of SP-A1 and 2 may also exhibit immune modulatory effects in diseases such asthma. We chose to study the SP-A2 variant with an amino acid substitution at position 223 based upon genotyping of a relatively small cohort of asthmatic participants with type 2 asthma. We did not compare peptide 223Q to 223K as we focused our 10 and 20-mer peptide experiments on the use of 223Q based upon our full-length SP-A and humanized mouse model data showing efficacy. Additionally, we acknowledge that our SP-A replacement/rescue experimental data may be slightly more compelling than the data from transgenic mice, which may be due to concentrations delivered to the source of inflammation given that we can give higher doses of peptides as opposed to the endogenous levels of SP-A that are expressed in the mice. Third, at this early time in development, we do not know the stability and half-life of our peptides in these model systems, which will be the focus of future studies. Finally, we also realize that inhibition of Stat3, as reported here and in our recent publication (4), may be one of many mechanisms, as SP-A is also known to exhibit immune modulatory functions in macrophages, eosinophils, mast cells and dendritic cells (21, 25, 26).

In summary, we report that SP-A exhibits genetic heterogeneity of function in models of HDM and IL-13 exposure. Using mouse models and primary human lung epithelial cells, a particular region of SP-A2 that includes position 223 has efficacy regarding inhibition of airway inflammation, MUC5AC gene expression and mucin production. Additionally, we provide evidence that our SP-A-derived peptides reduce one of the key features of asthma, a reduction in bronchoconstriction to methacholine challenge, in two mouse models. We have evidence that SP-A does this through interactions on two key cell types: eosinophils and epithelial cells. SP-A induces eosinophil apoptosis which is in line with the reduced eosinophilia detected in the peptide treated mice in the HDM model (22). Furthermore, SP-A works through a newly discovered receptor on eosinophils for this activity, MYADM, which may also be the therapeutic target for the peptides in the HDM model (27). SP-A works directly on epithelial cells to reduce STAT-3 signaling, which may also be true of the SP-A peptides, however additional testing is needed (4). A reduction in STAT-3 signaling leads to less inflammatory cytokine production and less mucus- key features we observe with our peptide treatment. While the effect of these SP-A2 peptides in the setting of oropharyngeal delivery of IL-13 and HDM and cellular exposure of IL-13 are interesting, more work is needed to determine if SP-A peptides delivered by aerosol are a viable therapeutic strategy for human disease.

## Data Availability Statement

The raw data supporting the conclusions of this article will be made available by the authors, without undue reservation.

## Ethics Statement

The studies involving human participants were reviewed and approved by Institutional Review Board at the University of Arizona. The patients/participants provided their written informed consent to participate in this study. The animal study was reviewed and approved by Institutional Animal Care and Use Committee at the University of Arizona.

## Author Contributions

DF, YW, CM, MC, KA performed experiments. DB and HK performed statistical analysis. MN, HC, DV provided reagents and helped with data interpretation. DF, YW, MK and JL conceived of experiments. DF, MK and JL wrote the manuscript. All authors reviewed the manuscript.

## Funding

This work was funded by NIH grants from NHLBI and NIAID and from the Arizona Biomedical Research Commission. HL125602 (Ledford), AI135935 (Ledford), AI125357 (Kraft, Voelker, Chu) and ADHS16-162519 (Kraft).

## Conflict of Interest

Authors JL and MK are co-founders of RaeSedo Inc, a start-up company with the goal of developing novel peptidomimetic based therapeutics derived from an active area of SP-A.

The remaining authors declare that the research was conducted in the absence of any commercial or financial relationships that could be construed as a potential conflict of interest.

## Publisher’s Note

All claims expressed in this article are solely those of the authors and do not necessarily represent those of their affiliated organizations, or those of the publisher, the editors and the reviewers. Any product that may be evaluated in this article, or claim that may be made by its manufacturer, is not guaranteed or endorsed by the publisher.

## Supplementary Material

Supplementary Figure 1 C57BL/6 negative controls. **(A)** BAL analysis for IL-13 model of all genotypes, **(B)** BAL analysis for HDM model, **(C)** lung function of WT males to methacholine challenge.

Supplementary Figure 2 Full Western blots for Stat3. Representative Western blot analysis of p-Stat3, Stat3 and b-actin.
