# Supplementary figures and images for: Small Peptide Derivatives Within the Carbohydrate Recognition Domain of SP-A2 Modulate Asthma Outcomes in Mouse Models and Human Cells
Source: Front Immunol. 2022 Jul 8;13:900022. doi: 10.3389/fimmu.2022.900022 (PMC9304716; doi:10.3389/fimmu.2022.900022)

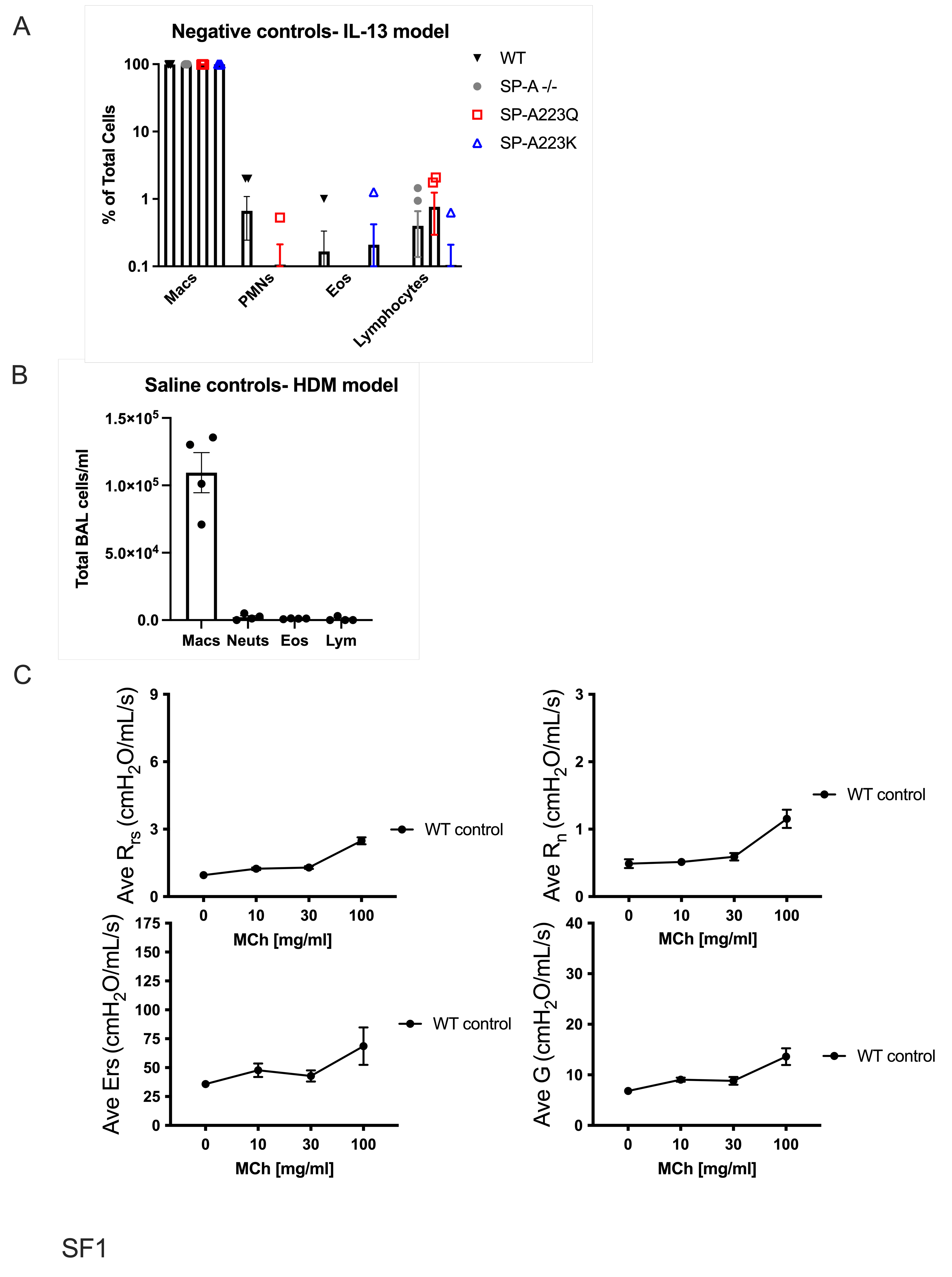

Supplement: Supplementary Figure 1 — C57BL/6 negative controls. (A) BAL analysis for IL-13 model of all genotypes, (B) BAL analysis for HDM model, (C) lung function of WT males to methacholine challenge. [file Image_1.tiff]

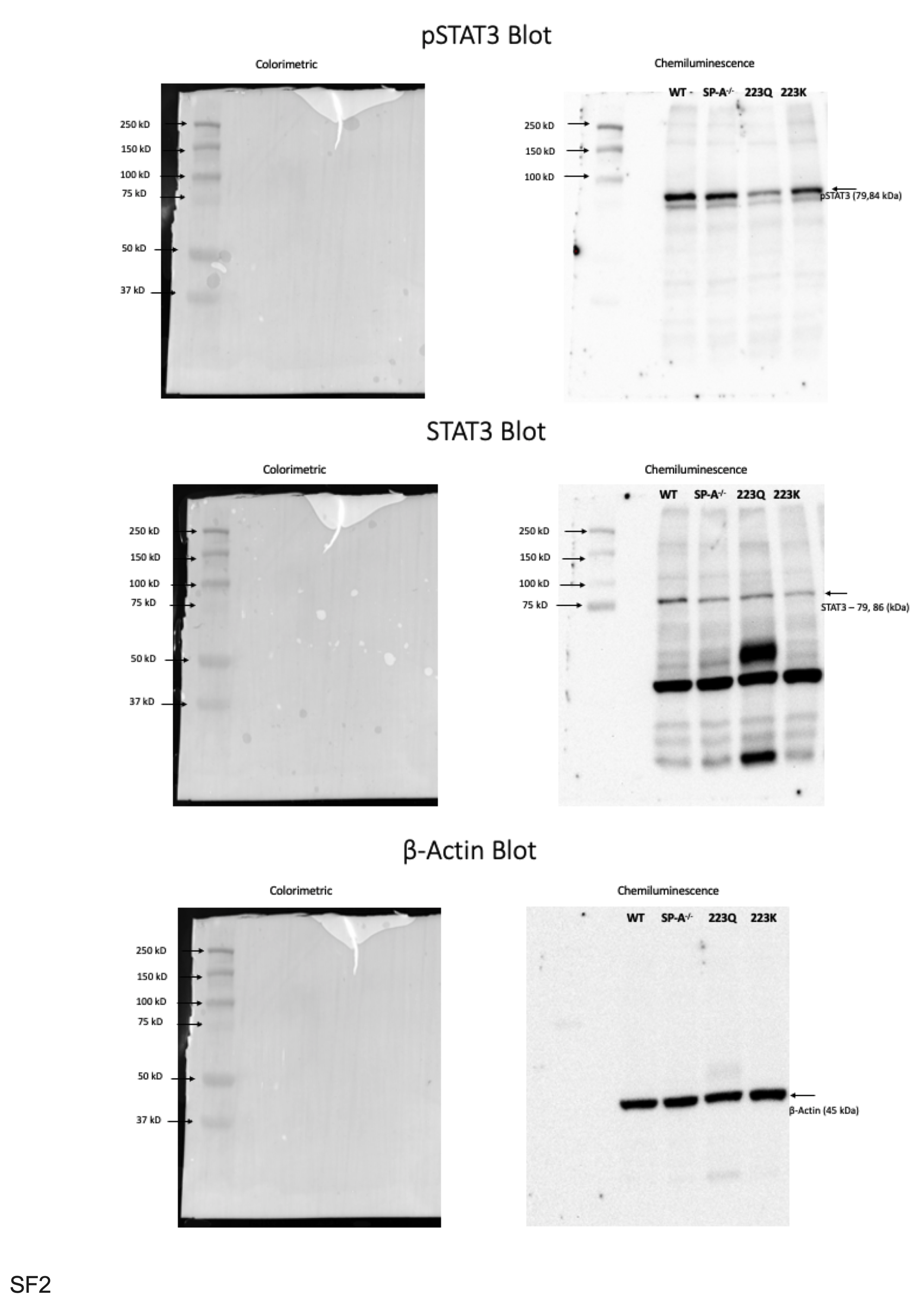

Supplement: Supplementary Figure 2 — Full Western blots for Stat3. Representative Western blot analysis of p-Stat3, Stat3 and b-actin. [file Image_2.tiff]
